# CXCL8_(3–72)_ K11R/G31P protects against sepsis-induced acute kidney injury via NF-κB and JAK2/STAT3 pathway

**DOI:** 10.1186/s40659-019-0236-5

**Published:** 2019-05-13

**Authors:** Yunfeng Zhou, Wenda Xu, Hong Zhu

**Affiliations:** 1 Department of Intensive Medicine, The Third Hospital of Nanchang, Nanchang, Jiangxi China; 2 Department of Intensive Medicine, Ruian People’s Hospital, No. 108 Wansong Road, Yuhai Street, Ruian, Wenzhou, 325200 Zhejiang China

**Keywords:** G31P, AKI, Inflammation, NF-κB, JAK2, STAT3

## Abstract

**Background:**

Acute kidney injury (AKI), which is mainly caused by sepsis, has high morbidity and mortality rates. CXCL8_(3–72)_ K11R/G31P (G31P) can exert therapeutic effect on inflammatory diseases and malignancies. We aimed to investigate the effect and mechanism of G31P on septic AKI.

**Methods:**

An AKI mouse model was established, and kidney injury was assessed by histological analysis. The contents of serum creatinine (SCr) and blood urea nitrogen (BUN) were measured by commercial kits, whereas neutrophil gelatinase-associated lipocalin (NGAL) and kidney injury molecule-1 (KIM-1) were detected by enzyme-linked immunosorbent assay (ELISA) kits. The expressions of CXCL8 in serum and kidney tissues were determined using ELISA and immunohistochemical analysis, respectively. Apoptosis rate of renal tissue was detected by terminal deoxynucleotidyl transfer-mediated dUTP nick end labeling (TUNEL) analysis. The expressions of inflammatory cytokines were measured by quantitative real-time PCR and Western blot, respectively. The apoptosis-related proteins, JAK2, STAT3, NF-κB and IκB were determined by Western blot.

**Results:**

G31P could reduce the levels of SCr, BUN, HGAL and KIM-1 and inhibit the renal tissue injury in AKI mice. G31P was also found to suppress the serum and nephric CXCL8 expressions and attenuated the apoptosis rate. The levels of inflammatory cytokines, pro-apoptotic proteins were decreased, while the anti-apoptotic proteins were increased by G31P in AKI mice. G31P also inhibited the activation of JAK2, STAT3 and NF-κB in AKI mice.

**Conclusion:**

These results suggest that G31P could protect renal function and attenuate the septic AKI. Our findings provide a potential target for the treatment of AKI.

## Background

Acute kidney injury (AKI), which is one of the severe complications induced by sepsis, is the most excessive systemic inflammatory response [1–3]. Though studies on kidney function have achieved a significant progress, the mechanisms of AKI still remain unclear and the mortality of septic patients is increasing [4]. Furthermore, AKI is demonstrated to be resulted from various factors including dysfunction and apoptosis of renal cells, intrarenal hemodynamic alterations and inflammatory infiltration in the kidney [5], among which renal apoptosis and inflammation are revealed to be critical causes to sepsis-derived AKI [6]. Recently, it has been reported that the pathogenesis of septic AKI was associated with inflammatory signaling pathways in addition to innate immunity [6]. As a crucial factor for health and diseases, apoptosis is proved to be able to cause kidney dysfunction in sepsis-induced AKI [7]. Moreover, apoptosis-related genes have been demonstrated to play a significant role in AKI and apoptosis was found more frequently in renal biopsies from patients with AKI who died from septic shock than those in controls [8, 9]. Therefore, it is imperative to find new effective therapeutic strategies.

It is shown that stimulated kidneys during sepsis could activate the nuclear factor-kappa B (NF-κB) signaling pathway to generate numerous pro-inflammatory cytokines including interleukin (IL)-6 and tumor necrosis factor-α (TNF-α) [9]. NF-κB, which was reported as a major target in a variety of diseases, plays a crucial role in maintaining the stability of the immune system [10–12]. Additionally, Janus kinase 2 (JAK2)/signal transducer and the activator of transcription 3 (STAT3) pathway are demonstrated to mediate the inflammatory response [13–15]. Furthermore, researches revealed that the JAK2/STAT3 pathway was involved in renal diseases [16–18]. The JAK/STAT signaling pathway is essential for immune and inflammatory responses. Previous study also showed that the expressions of TNF-α and IL-6 were increased by activated JAK/STAT pathway in rat lung tissue with sepsis [19], and that suppressing the JAK/STAT pathway could alleviate organ dysfunction in septic rats [20]. Thus, reducing inflammation by preventing the activation of JAK/STAT signaling pathways may have clinical benefits for patients who suffer from septic shock.

Chemokine (C-X-C motif) ligand 8 (CXCL8/IL-8), which can bind to G-protein coupled receptors (CXCR1 and CXCR2), is a chemokine mainly involved in the recruitment and activation of neutrophils [21]. CXCL8 contributes to the pathologies of angiogenesis, fibrosis, infection, arteriosclerosis, and tumor growth [22–26]. Besides, CXCL8 has been reported to be related to AKI in various diseases among patients [27–30]. CXCL8_(3–72)_ K11R/G31P (G31P), which is a CXCL8 antagonist with a more intimate connection to CXCR1 and CXCR2 than to CXCL8, is reported to suppress the chemotactic responses of neutrophils and inhibit the inflammatory response induced by neutrophils [31]. Moreover, G31P is revealed to attenuate cisplatin-induced nephrotoxicity, mitigate pathological kidney injury and alleviate infiltration of neutrophils in kidney [32]. However, the effect of G31P on the sepsis-induced AKI has been hardly reported, thus, our study aimed to explore the potential role in the sepsis-induced AKI.

## Methods

### Animals and ethics statement

Male C57BL/6 mice (8-week-old) were provided by The Third Hospital of Nanchang (Nanchang, China). All animals were acclimated for 1 week in a 12-h light/dark metabolic cage at room temperature with free diet and water provided. In our study, all animal experiments were approved by the Institutional Animal Care and Use Committee of The Third Hospital of Nanchang.

### Animal model and grouping

Mice were randomized into 5 groups (n = 10) as follows: control group, sham group, CLP group, control plus G31P group, CLP plus G31P group. CLP was performed as previously described [Rittirsch, 2009 #47]. An abdominal incision (1 cm) was made to expose the cecum, which was ligated with a 4-0 silk suture (5 mm) from the base of the ileocecal valve. Next, the cecum was doubly perforated using a 21-gauge needle. A small amount of stool was extracted from both holes, and the cecum was returned to the abdominal. The abdomen was then closed in two layers, followed by a subcutaneous injection of resuscitative normal saline. The mice in CLP group and CLP plus G31P group were administrated intraperitoneally with G31P (Institute of Biotechnology, National Tsing Hua University, Hsinchu, Taiwan) once every 2 days at a dose of 0.5 mg/kg. The vehicle group was treated with PBS, and the sham group received the same operative procedure, except for the colon ligation and puncture. The kidney tissues and blood samples were obtained for further analysis at 6, 12 and 24 h after CLP.

### Measurement of serum creatinine (SCr) and blood urea nitrogen (BUN)

The blood was centrifuged (3000 rpm for 10 min at 4 °C) at each time point (6, 12 and 24 h) after CLP. The levels of SCr and BUN, which were important indexes of renal injury, were detected following the protocol (Institute of Jiancheng Bioengineering, Nanjing, China) with an AutoAnalyzer (Roche Diagnostics, Mannheim, Germany).

### ELISA of urinary NGAL, KIM-1 and serum CXCL8

The expressions of urinary NGAL and KIM-1 and serum CXCL8 in different groups of mice were detected by corresponding Enzyme-linked immunosorbent assay (ELISA) kits (R&D Systems, Minneapolis, MN). The mean absorbance was detected at 450 nm on a microplate reader (Thermo Scientific, Pittsburgh, PA).

### Hematoxylin–eosin staining analysis and immunohistochemistry (IHC)

Kidney tissues in each group were fixed in 4% paraformaldehyde and blocked in paraffin. Thereafter, the sections were stained in hematoxylin for 5 min and eosin for 3 min. Images of HE sections were observed under an inverted microscope (Leica, Germany). 5 fields were randomly determined from each section. The kidney damage was evaluated according to the loss of brush borders (0–3), tubular vascularization (0–3) and inflammatory cell infiltration (0–3) [Zhou, 2014 #48]. All sections were independently assessed by an investigator in a blinded manner. IHC was performed as previously described [33]. To be more specific, the sections were washed by PBS and blocked in 3% H_2_O_2_ for 10 min. and were then incubated with goat serum for 15 min and washed. Subsequently, the sections were incubated with anti-CXCL8 (1:500, Abcam, Cambridge, UK) overnight at 4 °C followed by being incubated with streptavidin peroxidase-conjugated second antibody (Zhongshan Golden Bridge, Beijing, China) for 1 h at 37 °C. Next, the sections were stained with 3,3′-diaminobenzidine (DAB, Zhongshan Golden Bridge) for 6 min and then rinsed and stained in hematoxylin for 30 s. The sections were dehydrated in gradient alcohol and sealed in neutral resins. The integral optical density (IOD) was calculated with Image Pro plus 6.0 software (Microsoft Media Cybernetics, Bethesda, MD, USA).

### Terminal deoxynucleotidyl transfer-mediated dUTP nick end labeling (TUNEL) assay

The sections were incubated at 60 °C for 20 min and were then deparaffinized in xylene twice. Next, the sections were washed in graded series of alcohol and rinsed with PBS. Apoptotic cells were detected according to the protocol of TUNEL kit (Roche, Mannheim, Germany), and apoptotic (TUNEL-positive) cells were quantified under 400× magnification.

### RNA extraction, cDNA synthesis and quantitative real-time PCR (qPCR)

Total RNA of renal tissues was isolated using Trizol reagent (Invitrogen, San Diego, CA, USA). To be more specific, renal tissues were homogenized in 700 μL Trizol reagent and then by 300 μL chloroform. The samples were then mixed for 5 min. After centrifugation (12,000*g* for 15 min at 4 °C), the supernatant was carefully drew into a new tube. An equal volume of isopropyl alcohol was added and incubated at room temperature for 20 min. Following the centrifugation (12,000*g* at 4 °C for 10 min), the supernatants were removed completely and the precipitate was washed twice by 75% ethanol. Finally, nuclease-free DEPC water was added to elute the RNA, and the concentration and purity were detected by Shimadzu UV-2550 UV–visible spectrophotometer (Suzhou, China). The cDNA was obtained by 1 µg RNA according to the High Capacity cDNA Reverse Transcription Kit (Applied Biosystems, Foster City, CA, USA). In brief, the RNA was incubated with 2× RT master mix containing 10× RT Buffer, 25× dNTP Mix, 10× RT Random Primers and MultiScribe™ Reverse Transcriptase at 25 °C for 10 min, followed by at 37 °C for 2 h and at 85 °C for 5 min. The expressions of IL-1β (forward: 5′-TGC CAC CTT TTG ACA GTG ATG AG-3′ and reverse: 5′-TGA TGT GCT GCT GCG AGA TTT-3′), IL-6 (forward: 5′-AGG ATA CCA CTC CCA ACA GAC C-3′ and reverse: 5′-GCA CAA CTC TTT TCT CAT TTC CAC-3′), TNF-α (forward: 5′-ACT CCA GGC GGT GCC TAT G-3′ and reverse: 5′-GTG AGG GTC TGG GCC ATA GAA-3′) and GAPDH (forward: 5′-GCC TTC CGT GTT CCT ACC C-3′ and reverse: 5′-CAG TGG GCC CTC AGA TGC-3′) were determined in the ABI 7500 real-time quantitative PCR system (Life Technologies, Grand Island, NY) under the following conditions: at 95 °C for 5 min, 40 cycles at 95 °C for 15 s, at 56 °C for 30 s. GAPDH served as an internal reference gene and the data were analyzed by the 2^−ΔΔCt^ method (Table 1).

**Table 1:** Sequences of primers used for quantitative real-time PCR assays

Genename	Forward (5′–3′)	Reverse (5′–3′)
IL-1β	TGCCACCTTTTGACAGTGATGAG	TGATGTGCTGCTGCGAGATTT
IL-6	AGGATACCACTCCCAACAGACC	GCACAACTCTTTTCTCATTTCCAC
TNF-α	ACTCCAGGCGGTGCCTATG	GTGAGGGTCTGGGCCATAGAA
GAPDH	GCCTTCCGTGTTCCTACCC	CAGTGGGCCCTCAGATGC

### Western blot

Renal tissues were washed by PBS and lysed in lysis buffer (Beyotime, Shanghai, China), which were incubated on ice for 30 min and oscillated for 30 s. After being centrifuged at 10,000*g* for 30 min at 4 °C, the supernatant was collected to measure the protein concentrations using BCA kit (Solarbio, Beijing, China). The proteins separated by the SDS-PAGE were transferred onto polyvinylidene difluoride membranes (GE Healthcare, Little Chalfont, United Kingdom). 1 h after blocking, the membranes were incubated overnight at 4 °C with primary antibodies as follows: Bcl-2 (1:500, ab59348, Abcam), Bax (1:500, ab32503, Abcam), pro-caspase-3 (1:500, ab32499, Abcam), cleaved-caspase-3 (1:500, ab49822, Abcam), JAK2 (1:1000, ab108596, Abcam), p-JAK2 (1:500, ab32101, Abcam), STAT3 (1:1000, ab119352, Abcam), p-STAT3 (1:500, ab76315, Abcam), IL-1β (1:1000, #31202, Cell Signaling Technology, Beverly, MA), IL-6 (1:1000, #12912, Cell Signaling Technology), TNF-α (1:1000, #3707, Cell Signaling Technology), NF-κB (1:1000, #8242, Cell Signaling Technology), IκB (1:1000, #4812, Cell Signaling Technology) and β-actin (1:10,000, #4967, Cell Signaling Technology). The membranes were then incubated with secondary antibodies. The bands were determined by a Molecular Imager VersaDoc MP 5000 System (Bio-Rad, Hercules, CA). The densitometry was determined with a Quantity One (Bio-Rad).

### Statistical analysis

Data were expressed as mean ± SD and were assessed by one-way ANOVA, followed by Tukey post hoc tests using Graphpad Prism 5 software (San Diego, CA, USA). A P < 0.05 was considered as a statistically significant difference.

## Results

### G31P protected renal function and alleviated the kidney injury

**Fig. 1:**
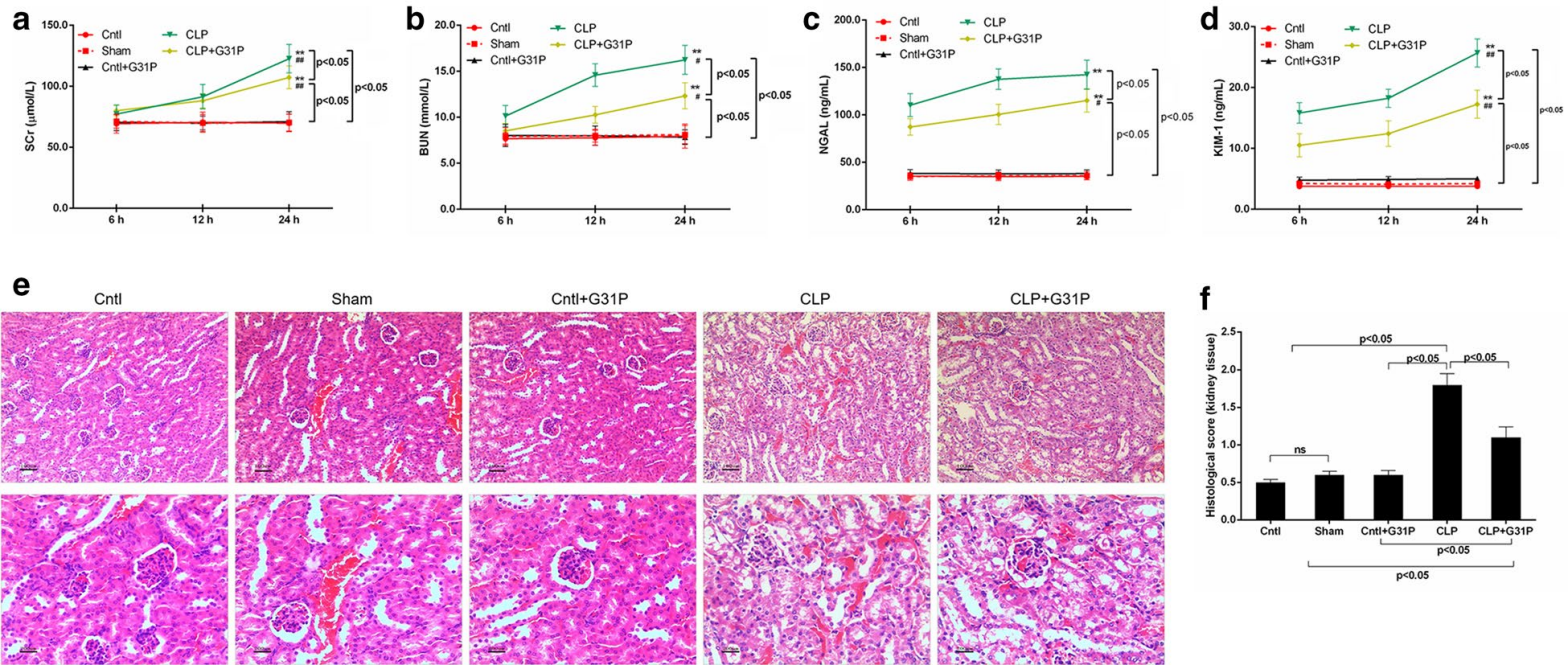
Effects of G31P on the renal function and kidney injury of mice. The blood samples of mice were harvested at 6, 12 and 24 h after the colon ligation and puncture, respectively. The levels of SCr (**a**) and BUN (**b**) were determined by chromatometry with kits while NGAL (**c**) and KIM-1 (**d**) were measured by ELISA. **e** Renal histological changes in mice were observed by H&E staining (magnification ×200). **f** The histological score of kidney tissue injury was evaluated by a blinded pathologist. ns represented no significant difference; ^★★^*P *< 0.01 versus the levels of biomarkers at 6 h in each group; ^#^*P *< 0.05, ^##^*P *< 0.01 versus the levels of biomarkers at 12 h in each group. G31P: CXCL8_(3–72)_ K11R/G31P; SCr: serum creatinine; BUN: blood urea nitrogen; NGAL: neutrophil gelatinase-associated lipocalin; KIM-1: kidney injury molecule-1

The role of G31P in kidney function was identified by the levels of several biomarkers including SCr (Fig. 1a), BUN (Fig. 1b), uric NGAL (Fig. 1c) and KIM-1 (Fig. 1d) at different time points. In CLP group and CLP plus G31P group, the levels of SCr, BUN and KIM-1 at 24 h after CLP were markedly elevated, compared with those at 6 and 12 h after CLP (P < 0.01). Similarly, the content of NGAL in CLP plus G31P group at 24 h after CLP was significantly more than those at 6 and 12 h after CLP (P < 0.05). While in CLP group, the NGAL level at 24 h after CLP had no obvious difference from that at 12 h after CLP. The levels of SCr and BUN were evidently improved 24 h after CLP, while the concentrations of uric NGAL and KIM-1 were went up significantly 6 h after CLP. Nevertheless, the contents of these biomarkers were significantly reduced by G31P, which were consistent with the histological changes (Fig. 1e). Obvious renal tubular changes such as abscission of tubular epithelial cells, glomerular hypertrophy, deprivation of brush border, loss of epithelial cell nuclei, prominent vacuolar transformations, epithelial cell necrosis and shed into tubular lumens were observed in CLP group. Moreover, the histological scores of kidney tissues were high after the CLP, while G31P could partly lower the scores (P < 0.05; Fig. 1f).

### G31P reduced the expressions of CXCL8 in renal tissues and serum

**Fig. 2:**
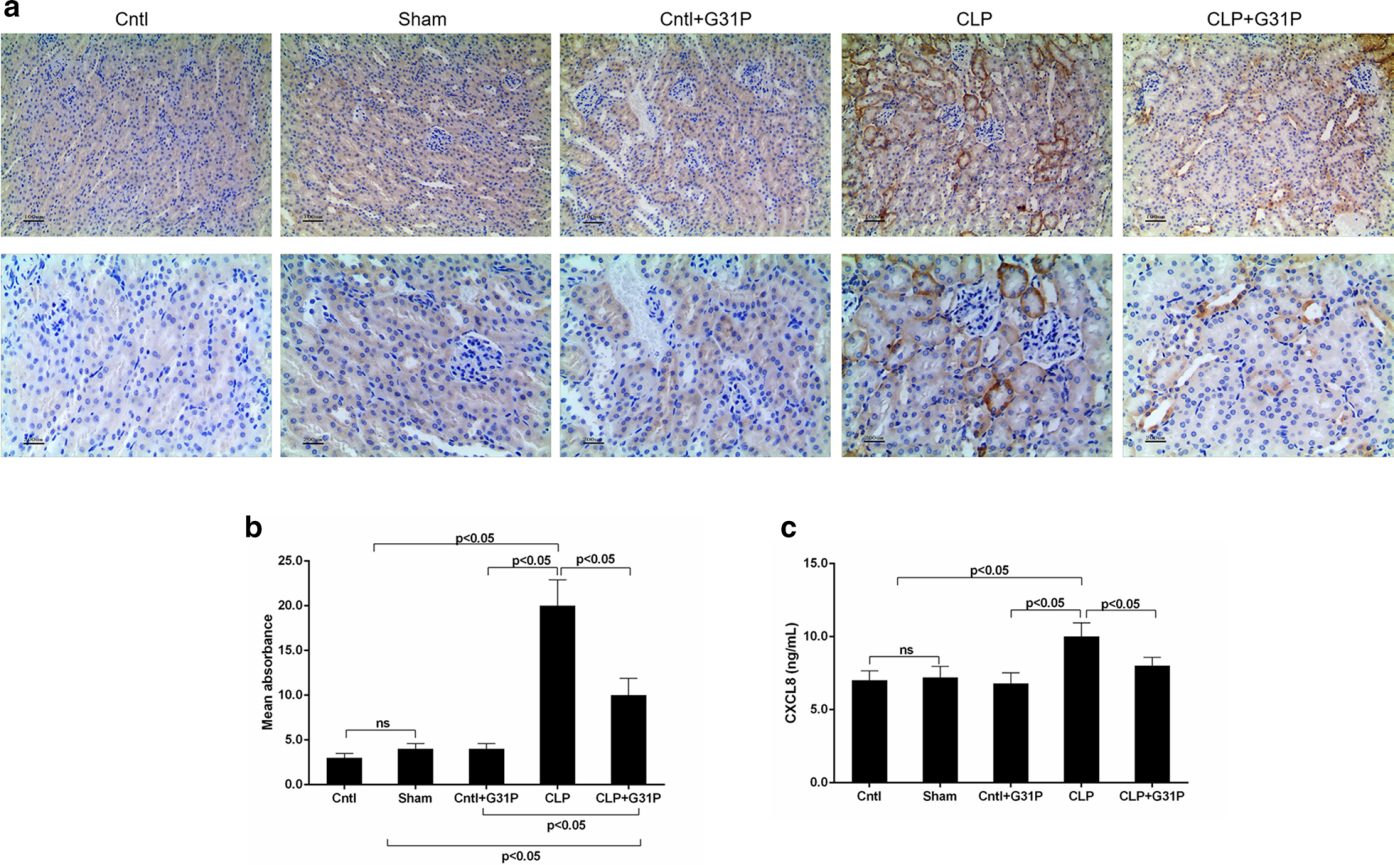
Effects of G31P on CXCL8 expression in mice. **a** The CXCL8 expression in renal tissues of mice in 5 groups was measured by immunohistochemistry (magnification ×400). The mean absorbance of serum CXCL8 (**b**) and the serum CXCL8 expression of mice in 5 groups (**c**) were determined by ELISA at 24 h after the colon ligation and puncture. ns represented no significant difference

The CXCL8 expression was significantly up-regulated by CLP and was inhibited by G31P in kidney tissues (Fig. 2a). We also found that the absorbance of CXCL8 in serum was similar to that in the tissues (P < 0.05; Fig. 2b), and that G31P evidently decreased the serum CXCL8 levels by approximately 25% (P < 0.05; Fig. 2c).

### G31P inhibited the apoptosis rate in renal tissues

**Fig. 3:**
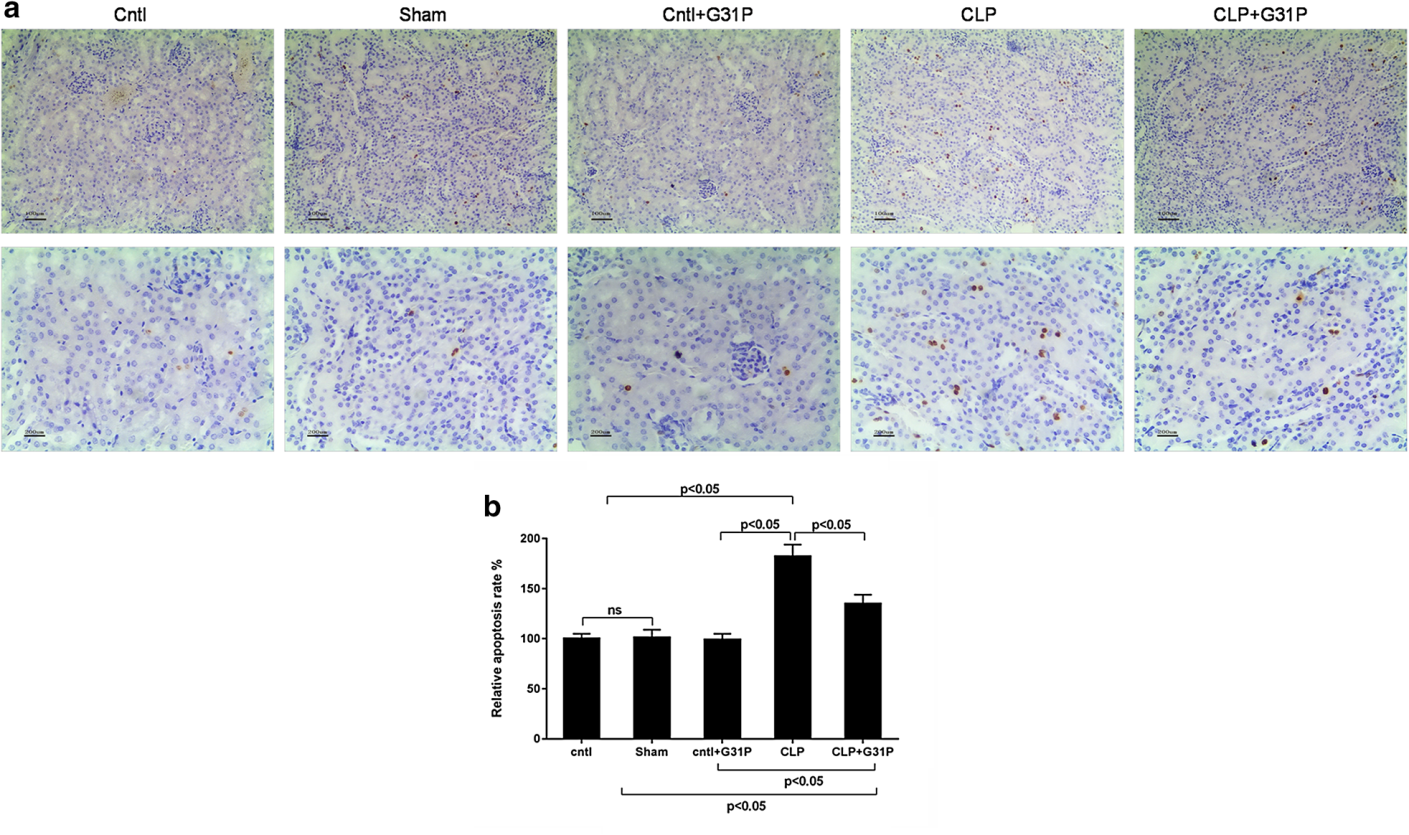
Effects of G31P on apoptosis of renal cells in mice. **a** The apoptotic cells (with brown nucleus) were detected by TUNEL analysis (magnification ×400) in 5 groups. **b** The related apoptosis rate of renal cells of the whole kidney. ns represented no significant difference

To determine the effect of G31P on apoptosis in septic AKI, the apoptotic cells of the kidney tissues were detected in the mice. The apoptotic cells were expressed as brown nucleus and apoptotic cells were found more in CLP group than those in sham group (Fig. 3a). Additionally, the apoptosis rate after CLP was increased, while G31P suppressed the apoptosis rate (P < 0.05; Fig. 3b).

### G31P exerted anti-inflammatory and anti-apoptotic effects in renal tissues

**Fig. 4:**
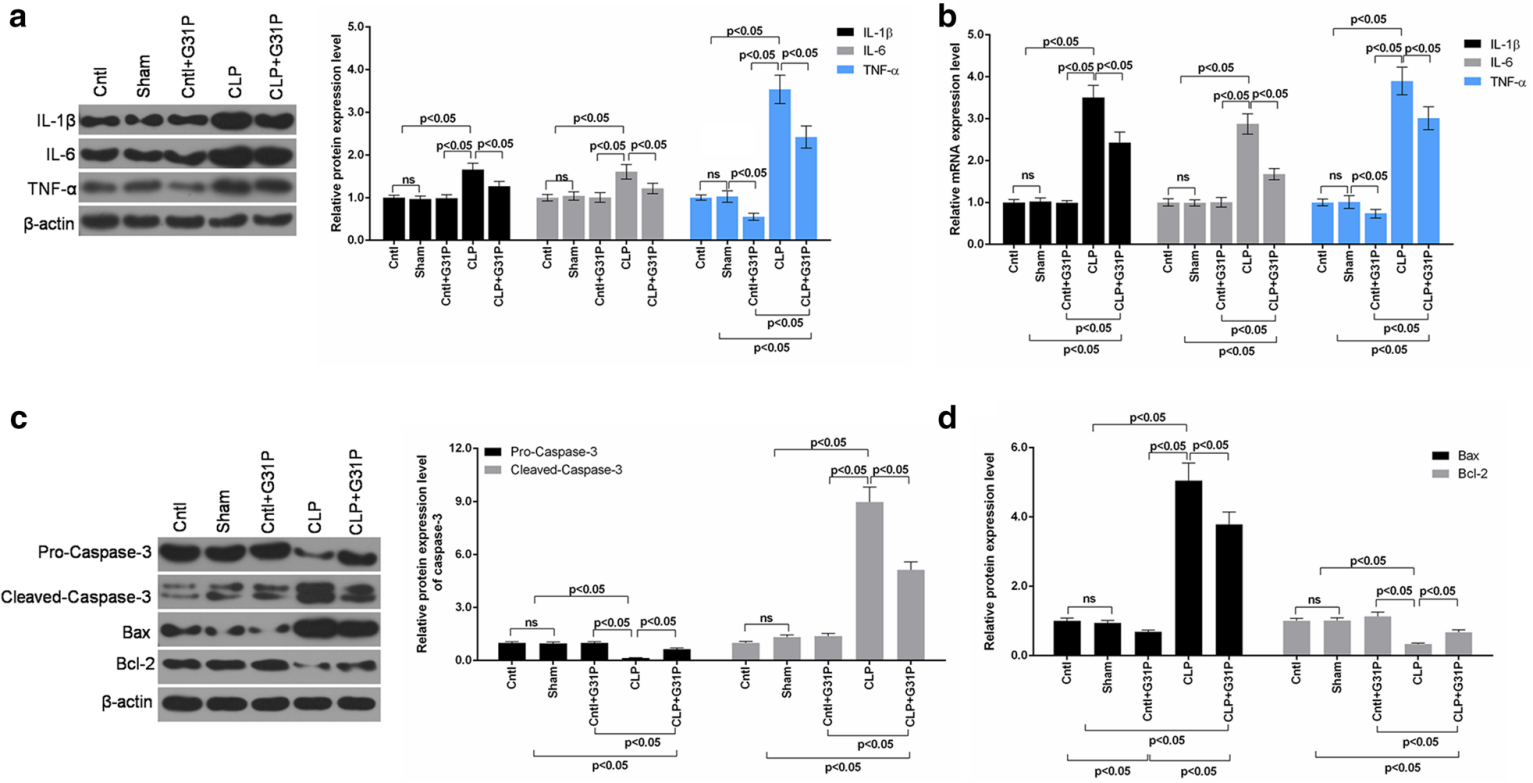
Effects of G31P on pro-inflammatory cytokines and apoptosis-related molecules of kidney in mice. **a**, **b** The protein and mRNA levels of IL-1β, IL-6 and TNF-α in 5 groups were determined by western blot and qPCR, respectively. **c**, **d** The expression of pro-caspase-3, cleaved-caspase-3, Bcl-2 and Bax of mice in 5 groups were detected by western blot. ns represented no significant difference

Chemokines and cytokines are involved in the progression of sepsis-derived AKI [4], thus, we measured the expressions of several pro-inflammatory cytokines in kidney tissues at protein (Fig. 4a) and mRNA levels (Fig. 4b). The expressions of IL-1β, IL-6 and TNF-α were remarkably raised after CLP but were noticeably suppressed by G31P in renal tissues of mice (P < 0.05). Interestingly, the expression of TNF-α in the cntl group plus G31P was significantly reduced, however, the mechanism was unclear.

Besides, the levels of apoptosis-related proteins in the kidney were determined (Fig. 4c). The biomarkers of advancing apoptosis, pro-caspase-3 and cleaved-caspase-3 were measured. We found that the pro-caspase-3 level was decreased, while cleaved-caspase-3 expression was elevated in the kidney of CLP mice (P < 0.05). However, G31P reversed such an expression pattern (Fig. 4c). Another hallmarks of cell apoptosis, Bcl-2 (anti-apoptosis) and Bax (pro-apoptosis) were also detected in the renal tissues (Fig. 4d). Decreased Bcl-2 expression and increased Bax level were found in the kidney of mice with CLP, however, the expression patterns were partly reversed by G31P (P < 0.05). Moreover, G31P inhibited the Bax levels of mice in the cntl plus G31P (P < 0.05).

### G31P suppressed the activation of JAK2 and STAT3

**Fig. 5:**
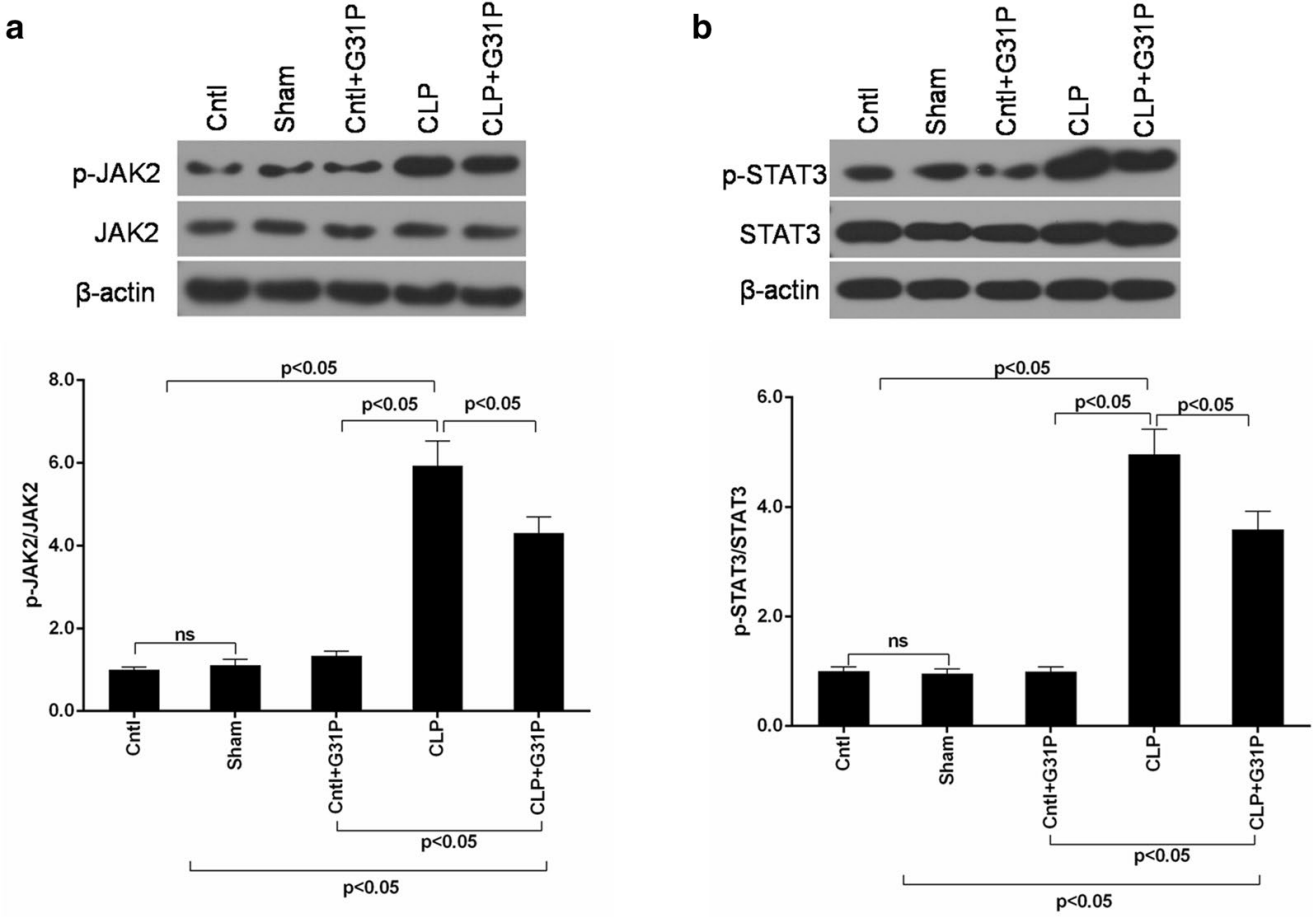
Effects of G31P on the expression of JAK2 and STAT3 in mice. **a** The protein levels of p-JAK2 and JAK2 in 5 groups were measured by western blot. **b** The protein expression of p-STAT3 and STAT3 of mice in 5 groups were measured by western blot. ns represented no significant difference

To investigate the effect of G31P on the phosphorylation of JAK2 and STAT, the protein levels of p-JAK2, JAK2, p-STAT3 and STAT3 were determined. The CLP treatment evidently increased the expression of p-JAK2, which was significantly abated by G31P (P < 0.05; Fig. 5a). Similarly, the level of p-STAT3 was markedly induced in the CLP mice but was also reduced by G31P (P < 0.05; Fig. 5b).

### G31P prevented the activation of NF-κB

**Fig. 6:**
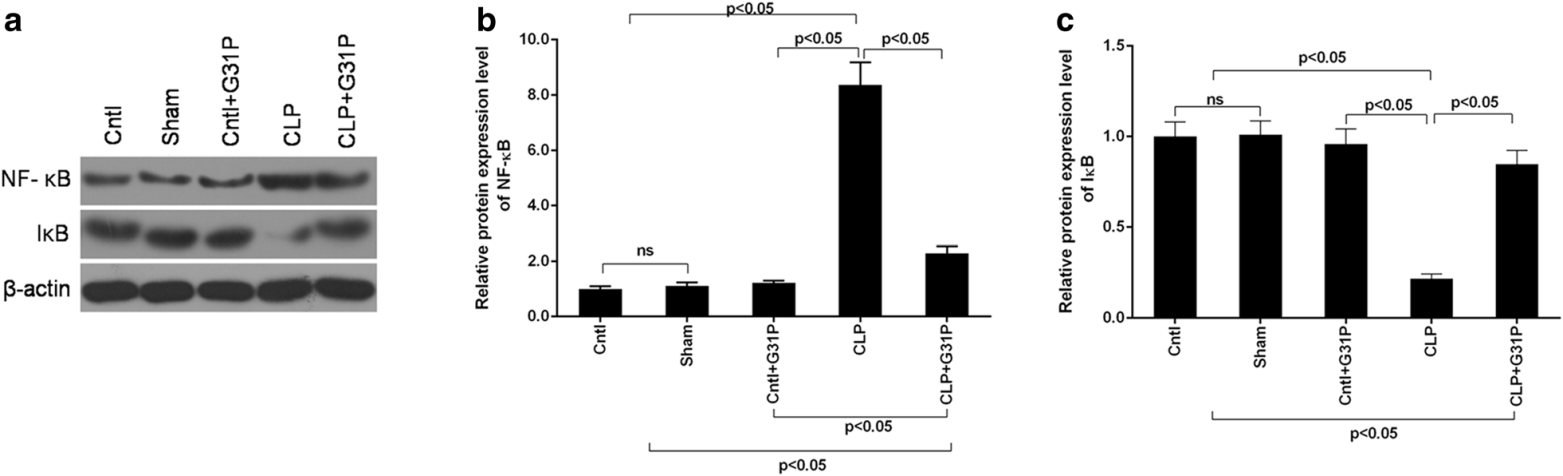
Effects of G31P on the expression of NF-κB. **a**–**c** The protein levels of NF-κB and IκB of mice in 5 groups were detected by Western blot. ns represents no significant difference

To explore the potential roles of G31P in the septic AKI, we detected the levels of renal NF-κB and IκB in mice (Fig. 6a). Though NF-κB expression was evidently higher in the CLP mice than that in sham group, its expression was suppressed by G31P (P < 0.05; Fig. 6b). Also, the decreased protein expression of IκB in CLP mice was recovered by G31P (P < 0.05; Fig. 6c).

## Discussion

As septic AKI has high morbidity and mortality [34], it is imperative to find effective therapeutic strategies for treating septic AKI. In the current study, we successfully established a sepsis-induced AKI mouse model. We found that G31P could protect renal function and attenuate the AKI by inhibiting the inflammation and apoptosis. Moreover, the JAK2/STAT3 and NF-κB signaling pathway might be involved in sepsis-induced AKI.

The SCr and BUN are biomarkers of renal dysfunction [35]. Additionally, many protein products have been found as novel markers such as NGAL and KIM-1 in the initial stage of AKI [36–38]. KIM-1 is difficult to be detected in healthy kidneys, however, it is highly expressed in proximal tubule cells after ischemic and nephrotoxic injury [39]. NGAL is shown to have a prognostic value in predicting acute and chronic nephropathies and deterioration of renal function in patients [40]. In our study, the levels of KIM-1 and NGAL were increased sharply 6 h after CLP and such an increase took place earlier than those of SCr and BUN, suggesting that these two biomarkers could ponder renal injury more accurately and faster than SCr and BUN [41]. Furthermore, G31P could reduce the levels of these biomarkers, which was in accordance with the histopathological alterations, indicating that G31P could improve the renal function and alleviate the kidney injury.

Pro-inflammatory cytokines can play a critical role in the later systematic inflammation response and subsequently multi-organ failure in sepsis [42]. CXCL8 is reported to recruit leukocytes from the blood into tissues during inflammation [43] and in turn, the inflammation deteriorated by activated leukocytes could increase CXCL8 level [44]. In the present study, the CXCL8 expressions in serum and kidney were up-regulated obviously in the CLP mice and were reduced by G31P, indicating that G31P might be involved in the inflammatory circle between inflammatory cells and chemokines. Furthermore, the CXCL8, which is a vital chemokine to trigger the local infiltration during renal inflammation, could be regulated by IL-1β and TNF-α [34]. Several pro-inflammatory cytokines have been demonstrated to play a critical role in septic AKI and other systemic dysfunctions in sepsis [45, 46]. Our results showed that G31P decreased the levels of IL-1β, IL-6 and TNF-α in the CLP mice, which was consistent with the results of the previous study on the role of G31P in the HFD/STZ-induced diabetic mice [44].

The apoptosis of renal tubular cells is another crucial pathogenesis of AKI [47]. In the current study, TUNEL analysis revealed that renal tubular cell apoptosis was increased after CLP, and this could be confirmed by the expressions of apoptosis-related proteins. Procaspase-3 can be activated by cleaving, and cleaved caspase-3 can lead to cell death [48]. Bcl-2 is an anti-apoptotic protein, while Bax exerts an opposite effect [49]. In our study, apoptosis was induced by increased expressions of cleaved-caspase-3 and Bax and reduced levels of pro-caspase-3 and Bcl-2 in CLP mice, which were reversed by G31P. These results demonstrated that G31P could inhibit the apoptosis in septic mice.

The activation of JAK2/STAT3 signaling is reported to be involved in the apoptosis and inflammation [50]. It has been reported that JAK2/STAT3 could induce mitochondrial mediated apoptosis [51] and regulate Bax expression in the ischemia reperfusion (IR) injury [52]. Recently, the JAK2/STAT3 signaling pathway has also been confirmed to play a crucial role in inflammatory responses [53, 54]. Furthermore, JAK2 and STAT3 can release of IL-1β, IL-6 and TNF-α and are involved in the inflammatory response, septic shock and acute organ injury [19, 55]. In our study, we found that the levels of phosphorylated JAK2 and STAT3 were enhanced after CLP. G31P could suppress the phosphorylation of JAK2 and STAT3 in CLP mice, suggesting that the JAK2/STAT3 pathway might be related to the anti-apoptotic and anti-inflammatory properties of G31P.

NF-κB is believed as an upstream regulator of inflammation to regulate numerous inflammatory factors. Once activated, NF-κB can deteriorate tissue injury by increasing several inflammatory cytokines [56]. The NF-κB signaling pathway can be activated by TNF-α and can aggravate the inflammatory response in AKI [57, 58], and the inhibition of NF-κB activity can attenuate septic AKI [59]. NF-κB can be released by phosphorylated IκB (inhibitory protein), and released NF-κB is phosphorylated and translocated into the nucleus to mediate the targets [60]. In the present study, an increased level of NF-κB and a decreased IκB expression were observed in the CLP mice. Additionally, the activation of NF-κB was suggested to be positively correlated to apoptosis [61]. Furthermore, the expression of caspase-3 is regulated by NF-κB in kidney tissues from septic patients [47]. In our study, the high level of NF-κB was inhibited, while the decreased IκB expression was improved by G31P in CLP mice, suggesting that G31P might also inhibit the activation of NF-κB, therefore exerting the anti-inflammatory and anti-apoptotic effects in mice.

## Conclusion

In conclusion, we have demonstrated that G31P could maintain the renal function and alleviate the sepsis-induced AKI by its anti-inflammatory and anti-apoptotic effects. Also, G31P can inhibit the phosphorylation of JAK2 and STAT3 and inactivate NF-κB after CLP. These results provide a potential therapeutic strategy for septic AKI.

## Data Availability

The analyzed data sets generated during the study are available from the corresponding author on reasonable request.
